# Network Governance at the Margin of the State: Rural Drinking Water Communities in Chile

**DOI:** 10.1007/s00267-022-01760-2

**Published:** 2022-12-07

**Authors:** Gabriela Estefania Bawarshi Abarzúa, Johannes Glückler

**Affiliations:** 1 Vicerrectoría de Investigación, Desarrollo y Creación Artística, Universidad de Chile, Diagonal Paraguay 265, Santiago, Chile; 2 Economic Geography Group, Institute of Geography, Heidelberg University, Berliner Straße 48, D-69120 Heidelberg, Germany

**Keywords:** Water, Network governance, Legitimacy, Collaboration, Social network analysis, Chile

## Abstract

We focus on the relationship between the network structure of Chilean rural drinking water associations (APRs) and effective governance outcomes regarding the provision of infrastructure and drinking water to peripheral rural communities in the Valparaiso region. Based on a comparative regional multi-method case study, we assess the coherence of differences in the governance network structure with the corresponding governance outcomes. Using qualitative interviews, participant observation, and a network survey of collaboration and legitimacy relationships among leaders of local APRs, we find that when isolated APRs establish collective organizations, they can generate better governance outcomes even without support from the state. We demonstrate that higher levels of collaboration as well as a more integrative distribution of legitimacy relations in the network are coherent with more effective governance outcomes. The findings suggest to strengthen social and organizational capacity at the local level of water governance in order to overcome the challenges of megadroughts and of a lack of public infrastructure in peripheral rural areas.

## Introduction

Being used for agriculture, mining, energy, recreation, and even spiritual purposes, water is one of the most precious natural resources. Its overexploitation constrains the recharge capacity of river basins and has changed some of the world’s main rivers to the point of total drought (Molle et al. 2010). The growing scarcity of water has aggravated the conditions of access to water in terms of quantity and quality, especially in relation to human and domestic consumption. Globally, four billion people live in conditions of severe water scarcity, at least 1 month a year (Mekonnen and Hoekstra 2016). A United Nations General Assembly resolution formally recognized the right to water and sanitation in July 2010. It acknowledges that safe and clean drinking water and sanitation are essential for realizing all human rights. Yet, the proportion of safely managed water service in the rural areas of Latin America and the Caribbean is 42% (World Health Organization 2019, p. 47). In addition, rural communities are more vulnerable to environmental transformations because they depend more directly on natural resources for their livelihoods: “80% of the population in extreme poverty also live-in rural areas” (De La O Campos et al. 2018, p. 10). Despite financial efforts at the national and international levels to secure the necessary drinking water supply, more than a quarter of the existing rural water infrastructure worldwide is in trouble, and is currently failing to supply in continuity and quality (Valcourt et al. 2020).

Apart from the heterogenous nature of water, political, economic, cultural, and ethical issues about who should be in charge of its management and administration incur major challenges for water governance. Hardin (1968) assumed that people would adopt the rationality of homo economicus and seek maximum individual utility with minimum effort. In Hardin’s view, the management of water or any other resource could not be solved by cooperation. Therefore, the only way to sustainably preserve the common good would be for an external actor, either the market or the state, to set limits and guide the behavior of users and their relationship with the resource. However, empirical evidence shows that actors can adopt and develop practices of collective action that are based on cooperation and pursue common goals that benefit all members and the environment (Ostrom 1990, 2002). How organizations work together at the local level is a key issue for effective governance. Effective governance refers to the ability of the involved actors to meet collectively pursued objectives and achieve corresponding outcomes (Börzel and Panke 2007). Scholars have begun to favor networks over hierarchies as an appropriate form of natural resource management (Berdej and Armitage 2016; Bodin and Crona 2009; Huang et al. 2020). To manage natural resources across geographic and political scales, many network governance initiatives have been established, e.g., in Montana, Alberta and British Columbia, to include more than 100 public, private and civil organizations in a space where they coordinate environmental conservation efforts (Bixler et al. 2016; Leong et al. 2011). However, we still lack knowledge about how networks operate to achieve effective governance outcomes and how such governance depends on the context and type of organization.

Chile ranks 18th on a global list of water-scarce countries (Rutger et al. 2019) and is the most water-stressed region in Latin America. In this paper, we examine how two communities responsible for rural drinking water systems compensate for the lack of public infrastructure to ensure water supply to their community. Rural drinking water organizations in Chile are called Asociaciones de Agua Potable Rural (APRs) and are dedicated to manage, maintain and operate water systems for surrounding families. These associations build and operate a small infrastructure that includes a water treatment plant, ponds, distribution networks and wells. We adopt the SONA approach—Situational Organizational Network Analysis (Glückler Panitz and Hammer 2020)—to combine formal social network analysis with semi-structured interviews to reconstruct and compare the relationships of two Communal Unions of rural drinking water associations^1^ in the region of Valparaíso, Chile. The empirical analysis will demonstrate how the structure of collaboration and legitimacy in lateral network governance influences governance outcomes in the absence of hierarchy. More specifically, the comparative case study suggests that the quality of governance among the local APRs was more effective than only the support by the state to attract financial resources, incorporate innovative activities, and build new knowledge to improve the physical infrastructure and build organizational bridges of coordination with other organizations.

## The Network Governance of Natural Resources

Governance may be conceived as a form of coordination that ranges between social institutions on one end and management on the other end of a duality of coordinating collective action (Glückler Herrigel and Handke 2020). Whereas institutions are long-term patterns of social order that are difficult to control or purposively change by individuals, management often refers to short-term decision making under the mandate of a legitimate authority. Governance falls between the two as it denotes a form of coordinating collective action, in which actors are interdependent rather than subject to a single authority, and in which actors negotiate interests and alternatives for action to pursue legitimate solutions to commonly shared challenges (e.g., Scharpf 1997; Jessop 1998; Peters and Pierre 1998; Rhodes 2007; Ostrom 2010). Such an understanding requires governance concepts that are able to address how actors affected by collective action dilemmas actually shape their actions and choose mechanisms and processes of governance to address governance objects in a given spatial context. The collective action dilemma in natural resource governance has been studied in depth in several related approaches (e.g., Ostrom 1990; Chaffin and Gunderson 2016; Folke et al. 2005; Schultz et al. 2019).

The governance of the commons, for instance, refers to a process in which a group of people shares the natural resource and where the mechanism of self-governance helps actors to achieve common goals (Ostrom 2010). Because decision making is based on a negotiation process between the actors involved (Bixler et al. 2016), this governance is characterized by bottom-up or lateral processes (Lazega 2000). Another aspect of commons governance is scale. While any coordination process implies the establishment and adherence to internal organizational rules, collective governance in most cases also requires coordination and decision making across different organizations and scales (Berkes 2008; Gruby and Basurto 2013). Ostrom (2010, 2014) proposed the concept of polycentric governance to implicitly allude to the notion of network. Polycentrism refers to the multiplicity of power centers that are interdependent and require constant coordination between them. This additional layer of complexity is also included in the concept of adaptive governance (Wyborn 2015; Urquiza et al. 2019).

The polycentric approach assumes that public, private and civic actors situated in the same governance context have an equal seat at the table. At the same time, because there are many controlling bodies, power is decentralized to multiple members, resulting in greater autonomy for self-governance. Scholars of political ecology have questioned the role of participation in this type of governance process (Bustos et al. 2019), because in reality actors often do not have equal power and their interactions are mediated by political or economic interests that do not involve all participants equally in decision making (e.g., Höhl et al. 2021). In this context, self-governance requires clear and acceptable agreements on participation, consultation, accountability, equity, and interconnection with other levels to maintain the legitimacy and resilience of the system being governed (Lebel et al. 2006). In the case of water, where a variety of actors and levels are involved in governance, the state must be strong enough to enforce reliable regulation by defending collective interests (Mayntz 2001) and sanctioning violations.

A related approach, adaptive governance (Schultz et al. 2019) focuses primarily on resilience, i.e., “the capacity of a system to absorb disturbances and reorganize itself as it changes so that it retains essentially the same function, structure, identity, and feedback” (Walker et al. 2004, p. 2). By learning how adaptation works in times of crisis, it is possible to build adaptive, sustainable and equitable network governance for natural resources. Consequently, the variety of approaches, including governance of the commons, adaptive governance, and polycentric governance, all focus on situations of delegated (across levels) and distributed (across organizations) networks and decision making.

In light of this debate, it still remains a pending question how particular patterns of governance relations convey effective outcomes in particular contexts. It is this relational context in which network governance approaches have begun to develop solutions for governance practice (Jones et al. 1997; Provan and Kenis 2008; Bodin and Crona 2009; Scott 2015; Keast 2016; Gutiérrez and Glückler 2022). To advance our understanding of the relational forms and processes of collaboration and legitimation in governance, we adopt the concept of lateral network governance (Glückler and Hammer 2015; Glückler 2020). Contrary to vertical forms of top-down governance, where hierarchy and authority outweigh participation, lateral network governance focuses on the micro-level of interactions among interdependent actors. A key assumption is that effective network governance depends on the distribution of legitimacy (Lazega 2000; Lazega and Krackhardt 2000; Bogason and Musso 2006). Decision making in networks can lead to effective and sustainable outcomes when found legitimate by others. Especially in conflicting and uncertain environments legitimate decisions are crucial (Adger et al. 2005; Höhl et al. 2021; Albornoz and Glückler 2020; Steelman et al. 2021). Legitimacy refers to what people regard as acceptable and appropriate, and it represents a key principle for institutions to be stable over time (Human and Provan 2000; Bathelt and Glückler 2014; Sandström et al. 2014). Actors attain a status of legitimacy either by being elected to formal office or by earning trust and reputation for consistent, reliable, and accountable behavior. Legitimacy is thus a transversal concept that goes beyond formal organizations and their governing bodies to include informal social relationships that also affect a member’s level of legitimacy in network governance. Legitimacy and collaboration are often interrelated and fundamental to maintaining the stability of a governance system. The concept of lateral network governance helps to understand how relational forms of cooperation and legitimacy are related to governance effectiveness. In the context of networks, effectiveness can be defined as “the attainment of positive network-level outcomes that could not normally be achieved by individual organizational participants acting independently” Provan and Kenis 2008, p. 230). In this case study, we seek to determine the governance structure of formal and informal collaboration as well as the pattern of legitimacy and to assess the extent to which certain characteristics of this structure are related to a set of intended outcomes.

## Governance at the Margin: Rural Drinking Water Systems (APRs)

### Water Scarcity in Chile

Chile has experienced a severe water scarcity for several reasons. First, a continuous decline in precipitation has led to an uninterrupted succession of dry years since 2010 (CR2 2015), which is often referred to as the so-called megadrought (Gutiérrez and Glückler 2022). Second, and concurrently, changes in land use from smallholder agriculture to extensive monocultures of various trees have increased water use by agro-industrial companies in the country’s many drylands (Budds 2012). Third, Chilean legislation, enshrined in the 1980 Constitution, has had a negative impact on water management and use (Bauer 2004). For example, the state awarded natural resource concessions that allow private ownership of water through water use rights (DAA). The Directorate General of Water (DGA) grants these rights of use in the form of benefits in perpetuity. These rights are non-transferable, so the authorities have little potential for intervention in the case of overuse (Bauer 2002). Finally, there is continuous illegal extraction of ground and surface water (Budds 2012). Together, these factors have resulted in two major rivers in the Valparaíso region (La Ligua and Petorca) of Chile drying up completely. The decline in groundwater levels has affected small farmers and several communities in the region that receive water for human and domestic use through the rural drinking water system, which is characterized by shallow wells with precarious infrastructure. This water shortage is also understood as a socially constructed scarcity, a concept that several authors have introduced to account for the fact that drought is not only a physical phenomenon, but also a social, political and economic one (Oppliger et al. 2019). Scarcity mainly affects rural populations living in dispersed locations, who lack adequate drinking water infrastructure to adapt to these crises and to ensure domestic water consumption (Ministerio de Obras Públicas 2012).

### APRs: Rural Drinking Water Associations

Because sanitation companies are economically unsustainable in rural Chile, cooperatives or civic committees have filled the supply gap by way of construction of wells in order to extract groundwater. These so-called APRs grew out of the Rural Drinking Water Program established in Chile since 1964 (Olbrich and Valencia 2017) to organize for local water supply and distribution. The program had been implemented in response to international pressure and the commitment to ensuring universal access to drinking water in rural areas. Today, the program is mainly in charge of the Ministry of Public Works (MOP). The MOP subsidizes the infrastructure necessary for APRs to operate and outsource through private sanitation companies to advise and train them (Donoso et al. 2015). The law authorizes the state to invest only in projects intended to provide, operate and maintain drinking water in rural areas. Additional investments, such as in sewage treatment, require the action of other organizations, e.g., municipalities or regional governments.

APRs are the only model in sanitation that is not run by private companies. Some researchers have criticized its management as being inadequate, given the constant interruptions caused by lack of water or water quality problems due to contamination of the source or difficulties in the chlorination process (Blanco and Donoso 2016). However, despite the structural inequalities of poverty in rural areas, the public-municipal alliance that characterizes the management of APRs has achieved a high percentage for covering concentrated rural areas. Ensuring maximum coverage and quality of care in semi-concentrated and isolated rural areas remains a major challenge. In these more dispersed rural areas, the state has not sufficiently invested in drinking water supply, and this situation is observed worldwide (Valcourt et al. 2020).

To fill the remaining supply gaps, citizens in the community have created new water supply organizations called non-MOP-APR (Ministry of Public Works, 2019). These are centralized civic water supply associations, which provide service to a much smaller number of families without the support and funding by the Ministry of Public Works. Therefore, non-MOP-APRs have to constantly seek alternative sources of funding to build and maintain local water infrastructure. In recent years, non-MOP-APRs have gradually been incorporated into the Rural Drinking Water Program to ensure compliance with the state’s technical and quality standards (Blanco and Donoso 2016). Both types of APRs, the official MOP-APRs and the stand-alone non-MOP-APRs, exist near the same rural areas and have had to cope with increasing water shortages. Today, more than 1.6 million people, i.e., 9% of the Chilean population, are supplied by rural drinking water associations (MOP-APR). Considering the same challenges and the fact that both types of associations (MOP-APR and non-MOP-APR) are located in the same area, they have decided to work together and form umbrella organizations, called Communal Unions, to pool their interests, resources and cooperation under a common leadership. In addition, these Communal Unions have actively engaged in fundraising and facilitated joint work on water infrastructure. Counterintuitively, some non-MOP-APRs have been more successful in achieving their goals than even official MOP-APRs. Understanding these differences in governance effectiveness requires deciphering the underlying practices and patterns of interaction.

In this study, we address the following research question: How are network characteristics of legitimacy and collaboration among APRs in a Communal Union related to governance outcomes? To analyze this question, we develop a comparative research design (section “Methodology”), to assess the differential governance outcomes and to examine the empirical consistency with the characteristics of the two governance networks (section “Results”).

## Methodology

### Study Area: Province of Petorca

**Fig. 1:**
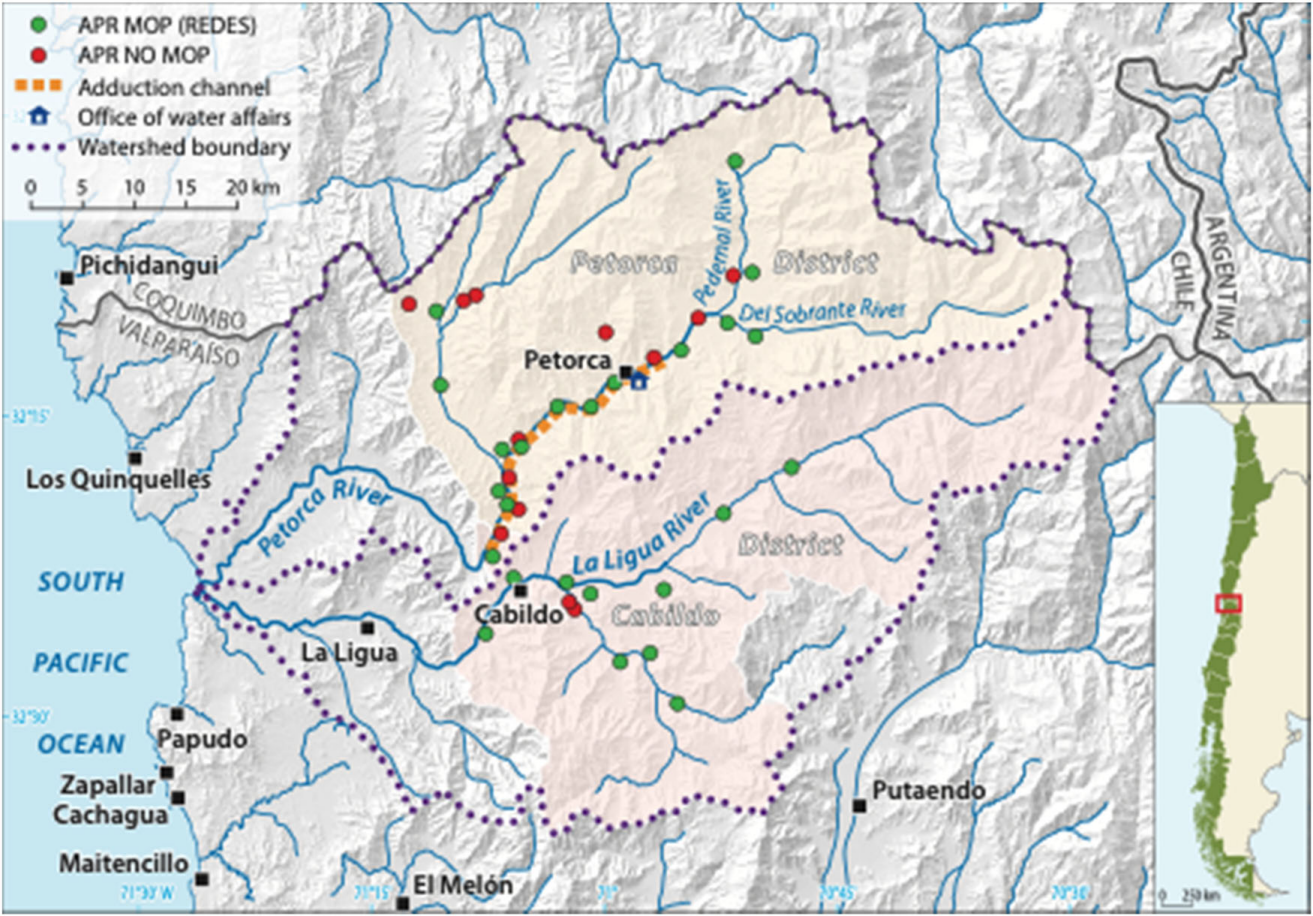
Distribution of APRs in the Cabildo and Petorca districts, Valparaiso Region, Chile (design by authors, cartography by V. Schniepp)

The water scarcity index is an international measure to determine the water gap as the difference between the total demand and actual supply of water (Zeng et al. 2013). According to this indicator, one of the most affected regions in Chile is Valparaíso. Moreover, a representative example of scarcity at the national level is the province of Petorca, a predominantly rural area in the Valparaíso region. It is divided into four districts: Petorca, Cabildo, La Ligua, Papudo and Zapallar (Fig. 1). It has an area of 4588.9 km^2^ and a population of approximately 79,000 people (INE 2018). This area received very little investment for even basic services such as education, health, and access to drinking water. The water crisis has caused problems in maintaining water supply to the population for more than a decade, which is reflected in successive declarations of scarcity decrees and emergency deliveries of water through truck transports (Miranda 2018). Within this area, we developed a comparative regional case study using mixed methods to compare two Communal Unions: Cabildo and Petorca. The APRs in these districts represent 51% of the total APRs in the province of Petorca. In both districts, leaders are organized in Communal Unions, a process that was mainly supported by leaders from both municipalities and external stakeholders.

### Data

To capture the contextual meaning as well as the formal structure of legitimacy and collaboration between APRs in both Communal Unions, we employed the integrative methodology of Situational Organizational Network Analysis, SONA (Glückler et al. 2020). It is one of several research strategies that responds to the call for more inclusive and meaningful relational research (Pachucki and Breiger 2010; Glückler and Panitz 2021). SONA involves a sequential application of qualitative and quantitative methods of data collection and analysis in a way that enriches theorizing and validating multiple sources of observations.

First, we conducted 25 exploratory interviews with APR leaders and local, provincial, regional and national organizations responsible for managing rural water supply: The National Federation of Rural Drinking Water, the Directorate General of Water (DGA), the Directorate of Hydraulic Engineering (DOH), academics from the University of Chile and Playa Ancha, social movements related to water, the Sanitary Company (ESVAL), several municipalities (Petorca, La Ligua, Cabildo), foundations and agricultural companies in the sector. Interviews were recorded and partially transcribed, and transcripts were imported into MAXQDA (VERBI Software, 2019) to conduct qualitative content analysis. Second, we conducted seven site visits to meet stakeholders at their monthly meetings and to build the trust necessary for them to participate in our network survey. Both the interviews and site visits were critical to understanding and specifying the levels of interaction, the specific activities in which they collaborated, and how legitimacy and collaboration would be observed in the subsequent network analysis.

Third, we conducted a standardized network survey among all APR leaders and external members of the two Communal Unions in Cabildo and Petorca districts. For external collaboration, we completed the network by collecting information on external collaborators at local, regional and national levels. The questionnaire included three sets of questions: the first related to respondents’ characteristics, including age, gender, qualification and employment status, role in the organization, level of involvement and sense of belonging to the Communal Union. The second set of items included three network-generating questions, one on legitimacy and two on collaboration (Table 1). We observed network legitimacy by surveying interpersonal relations for legitimate delegation of decision-making authority (Glückler 2020): respondents were asked to whom they would delegate decision-making authority (legitimacy) if they could not personally attend such a meeting. In addition, we observed collaboration by asking respondents to indicate both internal and external organizations with whom they frequently collaborated in maintaining and improving the APR system (Table 1). The third set of questions addressed the organizational level of the APRs, including their status as either a state-supported MOP-APR or non-supported non-MOP-APR, year of establishment, number of households served with water, major organizational and infrastructural problems, current water supply situation, drought management measures, and current water prices.

**Table 1:** Measures of collaboration and legitimacy between members of APRs

Item	Type of relation	Survey question
Internal collaboration	Mutual aid actions, to maintain the APR system	“During the last two years which of these APRs has directly helped you to carry out activities that have enabled you to operate and maintain the quality of the APR service (…)?”
External collaboration	Organizations involved in water governance providing resources to APRs	“During the past two years, which of these organizations has directly assisted you in obtaining information, technical, financial and administrative resources?”
Legitimacy	Delegation of decision-making authority	“Imagine that an important decision has to be made at the communal union meeting (around the issue of APRs) and you cannot participate in it, which other person from the communal union should not be missing to defend your point of view when making a final decision?”

The survey was realized in the midst of the COVID-19 pandemic, between June and July 2020. Although we collected most responses in face-to-face interviews during our fieldwork, some interviews had to be done by phone. In the case of Cabildo, all 16 APRs responded to the questionnaire, whereas in Petorca 22 (88% response rate) APRs completed the questionnaire, making up for 38 out of 41 APRs in the two districts. We further supplemented the survey data with information on the main technical problems and subsidies for each APR from the public “Water Plan for Petorca”. We used the program R and the network analysis suite implemented in it to construct, map and analyze the network data obtained from the survey.

### Measures

Our analysis focuses on the structure of the governance network of the Communal Unions in terms of two relationships: collaboration and legitimacy. We adopted the legitimacy measure from the lateral network governance model (Glückler 2020) and tailored the items to the specific context of APRs in Petorca province (Table 2). For both relations—collaboration and legitimacy—we calculated the following network measures:

**Table 2:** Subsidies received by the two Communal Unions, 2018–2020

	Cabildo	Petorca
Number of households	7037	7425
Subsidies received in million CLP	$1012	$2100
Number of APRs in the district	15	23
MOP-APR (share)	73%	60%
Non-MOP-APR (share)	27%	40%

#### Strength

At the individual level of APR leaders, we used indegree centrality as a measure of the magnitude of an individual’s legitimacy in the network. This measure counts for each member the number of votes that this member receives from their peers. At the network level of Communal Unions, we computed density as the proportion of all reported relations between the APR leaders of a Communal Union and the maximum possible number of relations. Density expresses the level of activity in the entire governance network. According to Bodin and Crona (2009), the higher the density of relations, the greater the potential for collective action. However, empirical evidence suggests that if network density is too high, the resulting homogeneity of actors may not allow for further development of learning and knowledge creation (Bodin and Norberg 2005).

#### Structure

The particular empirical distribution of legitimacy and collaboration relations constitutes a specific network structure, which we assessed by using two measures. First, indegree centralization measures the tendency for all relations in a network to concentrate on a single actor, and its values vary between zero and one. Centralization is one if all votes by all actors are directed to one single member, a situation that corresponds with a star image of a network graph. On the other extreme, centralization is zero if every member sends and receives the same number of votes (Wasserman and Faust 1994). Second, we adopted the legitimacy matrix (Glückler 2020) to assess whether members gain legitimacy locally, within a faction or subgroup of the network, or globally from across the different regions of the network. To calculate local and global legitimacy, we used cluster analysis to determine the number and composition of factions in each Communal Union network. A faction is a subgroup in which each member is more connected inside the faction than to actors outside that faction. Once the factions were identified, we used the External-Internal Index (Krackhardt and Stern 1988) to calculate the tendency for each APR leader to receive legitimacy nominations from within or across the factions in the network. The EI-index varies between −1 = relations exist only within a faction, and +1 = relations exist only across different factions.

## Results

### Variation in Governance Effectiveness

Despite many similarities, the two Communal Unions differ greatly in terms of governance effectiveness. Network effectiveness expresses the achievement of collectively intended outcomes at the level of a Communal Union (an association of APRs in an area) that would be difficult to achieve if APRs acted individually rather than collectively (Provan and Kenis 2008). Independent of their status as MOP or non-MOP, all APRs rely on external resources, whether from local, regional or provincial governments, private companies or foundations (Olbrich and Valencia 2017). In addition, the state enters into agreements with sanitation service providers to offer technical, administrative, organizational, financial and community guidance to APRs through the Technical Unit (UT) (Blanco and Donoso 2016). The main difficulty is that these advisory services are only provided to the organization that reports to the state. As for the case study, there were a total of 74 APRs in the province of Petorca, 43% of which did not belong to MOP and consequently did not receive any financial assistance or training (Ministerio de Obras Públicas 2019). Almost half of the APRs in the sector were in a precarious situation regarding water infrastructure and management. Severe droughts caused these systems to be unable to provide a continuous supply of potable water to the community, and the state had invested exclusively in MOP-APRs. An intuitive conjecture would suggest that the Communal Union with a higher share of MOP-APRs, i.e., those that receive subsidies through the top-down governance system, is likely to be more effective than a Communal Union with a minor share of MOP-APRs.

We observe governance effectiveness in two ways. First, we use the quantitative measure of the total amount of subsidies received by each Communal Union during the years 2018–2020. Second, we also assess a qualitative dimension by observing the quality of the collective activities and practices carried out by the actors of each Communal Union. Lack of financial resources is one of the most significant difficulties any rural drinking water association has to face. The infrastructure to build infrastructure to store, transport, and extract water is costly, so it is essential to take the necessary steps, coordinate efforts with other actors and generate advocacy to obtain the subsidies the state provides. We compile the data from the Water Plan for Petorca, which lists the investment initiatives completed by each MOP-APR. Although the proportion of MOP-APRs in Petorca was lower than in Cabildo, its Communal Union received about twice as many subsidies as Cabildo (Table 2). Although the granting of these subsidies was determined by the state, the actual receipt of these amounts depended on the capacity of each organization and their ability to work with the appropriate agencies to find solutions to their technical and infrastructural needs. In the following sections, we seek to account for the difference in governance effectiveness by assessing the quality (section “Relational Quality of the Governance Networks”) and the structure (section “Relational Structure of the Governance Networks”) of both governance networks. Quality is assessed based on the collective practices identified as relevant in the governance literature. The structure is evaluated based on the legitimacy and collaborative relationships between the members of each communal union.

### Relational Quality of the Governance Networks

To understand the variation in terms of governance effectiveness, we first used the qualitative interviews as part of the SONA methodology to analyze the quality of interactions in the Communal Unions in each locality. We build on six important aspects identified in the literature on governance effectiveness (e.g., Westley et al. 2013; Khosravi et al. 2019; Hahn et al. 2006; Cooper and Wheeler 2015; Berdej and Armitage 2016; Ulibarri and Scott 2017; Ansell and Gash 2008), to code and categorize the quality of activities in the interview transcripts. These six features are innovation, knowledge, bridging organizations, sense of belonging, reciprocity, and common objectives. Each aspect refers to a set of relational practices, which we identified to be crucial in the local context of water governance in the Province of Petorca. Our assessment of these relational qualities varies considerably between the two Communal Unions (Table 3).

**Table 3:** Activities and relational quality of governance in the two Communal Unions

Criteria	Cabildo	Petorca
Innovation		
New activities, capacitation and awareness to save water	x	x
Establishment of parameters by consumption	x	x
Penalties for overconsumption of water	x	x
Creation of “ATLAS Hídrico^a^”, the APRs are registered with their needs		x
APRs connected through WhatsApp	x	x
Knowledge		
Help partner APRs to learn how to chlorinate, use the electric panel, lend pumps and wells, carry out administrative procedures and apply for funds or bonds		x
DOH validation of non-scientific instruments used by the community to search for water, such as dowsing rods	x	x
Bridging organizations		
Creation of the Water Affairs Office		x
Creation of an extensive water pipeline (“Aducción Hierro Viejo”) that connects and distributes water to nine APR systems		x
Creation of a department (“Hydrodynamic Node”) to improve the use and care of the Petorca watershed		x
Sense of belonging		
Monthly meetings, active participation among most of the leaders		x
There is no relational division between MOP and non-MOP-APRs		x
Social meaning of been a APR leader	x	x
Young population joining the leadership of the APRs		x
Reciprocity		
Community activities to raise funds; water “minga^b^”, raffles, workshops, etc.		x
Establishment of the notion of “water family^c^”		x
Shared agreement to prioritize consumption over agriculture in water supply		x
Common objectives		
In search of common and enduring solutions for domestic water consumption		x
Awareness of shortages due to overexploitation of agricultural enterprises	x	x
Long-term vision of establishing a provincial Rural Drinking Water Federation		x

^a^“ATLAS Hídrico” was a project generated by the Petorca RPA Communal Union and other actors to generate a cadastre of all the RPAs in the district’s basin in order to know their current status and evaluate their particular needs

^b^“Minga” is a pre-Columbian tradition of voluntary community or collective work for social or reciprocal purposes, currently in force in several Latin American countries

^c^The communal union of Petorca calls itself the water family, due to its characteristics of mutual help among leaders. See https://porlatierra.org/casos/208

First, innovation is critical to help systems evolve and become more resilient (Westley et al. 2013). Both Communal Unions implemented new measures such as establishing payment criteria based on the amount of water consumed per family, and penalties for overconsumption of water. Second, due to low levels of formal education most APR leaders lacked the technical knowledge necessary to managing an APR. Such lack of knowledge implied that APRs did not always fully comply with the legal regulations established by the state. Petorca, unlike Cabildo, built a bank of tools and materials needed to operate an APR, generating voluntary training among the leaders themselves. Another problem occurred in the confrontation of the traditional and local knowledge applied APR leaders over many years with the expert knowledge brought in by technical experts from public authorities. Our interviews revealed that the public authorities have tried to moderate these conflicts by respecting and using local knowledge of the Communal Unions to find new water wells in all the region. Third, Petorca created several bridging organizations that contribute to network governance by engaging with external organizations and facilitating access to new resources (Berdej and Armitage 2016; Heinze et al. 2016). In contrast, the Communal Union in Cabildo failed to create such external links. The evidence again points to the necessity for local network governance to connect with other levels and resources outside the immediate horizon in order to mobilize resources for improvement. The importance of bridging organizations resonates with evidence in the literature that innovation and development depend on the rewiring of multiple relations (Panitz and Glückler 2017) and the linking with unconnected networks (Valdivia and Delgadillo 2013). Fourth, Petorca APR leaders reported to share a sense of responsibility for water supply in a community and call themselves a “water family”. This gave APR leaders in Petorca a sense of reciprocity and collaboration because actors knew they had a common problem to be solved (Ansell and Gash 2008). Finally, in Petorca, members established a long-term vision of common goals, that was not shared in Cabildo. Overall, the Petorca Communal Union was clearly more active and had developed a better quality of activities, relationships and outcomes. We refer to outcomes as a mutual sense of belonging and reciprocity; the pursuit of common objectives; increments in knowledge and innovation; and coordination with other institutions through the creation of bridging organization (Table 3).

### Relational Structure of the Governance Networks

**Fig. 2:**
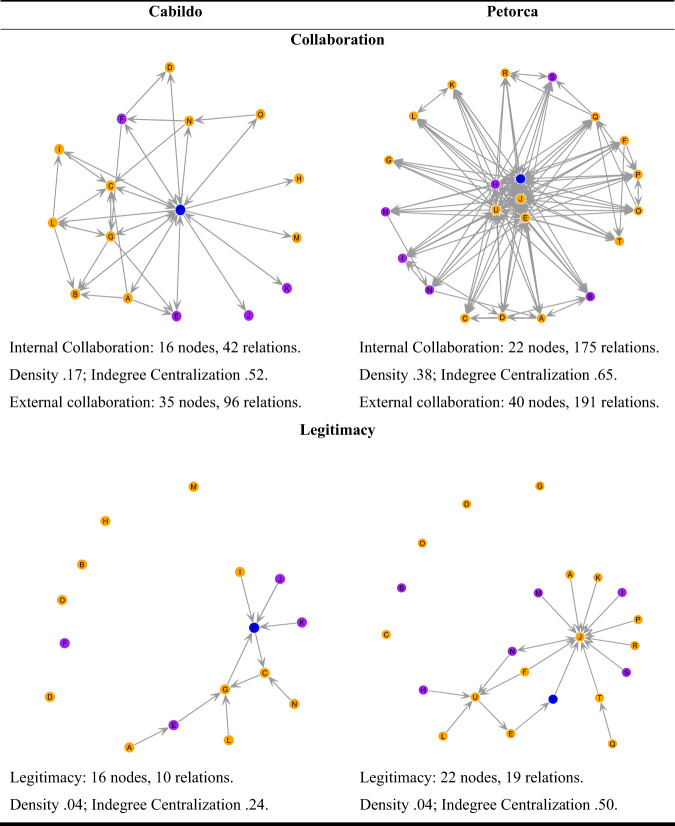
The network structure of collaboration and legitimacy in Cabildo and Petorca. Note: yellow = MOP-APRs; purple = non-MOP-APRs; blue = external organizations

The relational quality of attitudes and activities identified in the qualitative analysis of interviews was one aspect of the network reality. In the next step we combine these insights with an assessment of the network structure which is recognized as important to explain differences in governance outcomes (Sandström and Carlsson 2008; Wossen et al. 2013). Figure 2 shows a visualization of the collaboration and legitimacy networks of the two Communal Unions.

Generally, we find densities for collaboration in both networks to be higher than for legitimacy. This suggests that both Unions had not yet managed to build longer-term trust and legitimacy among their APR representatives at the same level that they had worked together and exchanged knowledge. This was owed to the fact that the Communal Union of Cabildo and Petorca had only established in 2015 and 2014. Both were still at an embryonic stage of organizational development, as one interviewee from an external organization argued (interview, Cabildo, August 2020). Statistically, we find collaboration and legitimacy to be highly correlated within the two Communal Unions (Cabildo: *r* = 0.84; Petorca: *r* = 0.69), suggesting that collaboration and legitimacy were mutually reinforcing and stabilizing the social relations and mutual commitments to common objectives. Furthermore, according to the indegree centralization, the collaboration networks tended to be relatively more centralized on a small number of key actors in both Unions. These central actors were also the ones that acted as bridging organizations to external partners.

Comparing the collaboration networks of the two Unions, we found that in Petorca, the most central actors included all the different types of organizations, i.e., MOP-APRs, non-MOP-APRs and external actors. Moreover, the Petorca Union had twice as many external relations as Cabildo, and maintained relations with organizations of many different scales. Empirical research as well as the theory of resource-dependence (Pfeffer and Salancik 1978) suggests that access to resources depends on the relations that organizations establish with other actors outside the networks across various hierarchical levels (Hahn et al. 2006). It can be inferred that support, innovation and organizational learning of Petorca’s APRs was more effective because its higher relational diversity facilitated connectivity with formally disconnected organizations (Sandström and Carlsson 2007). Whereas homogeneity may decrease resilience in the long run (Holling 2013), relational diversity supports learning and improve adaptability in the face of change (Urquiza and Cadenas 2015).

Communal Unions are organizational bodies that correspond to a form of network governance with no formal hierarchy in the decision-making process. As decisions are made by assemblies, participation is crucial to reduce the “noise” (Jessop 1998) that refers to the existence of different rationalities that compete in terms of their vision of governance. If actors failed to find a common language, it would be difficult for a Communal Union to thrive in the long run. Therefore, actors are themselves responsible for making and enforcing joint agreements. Evidence shows that building trust and legitimacy is especially fundamental for organizations to adapt to contexts of water scarcity (Schultz et al. 2019). Because the density of the legitimacy network was rather low in both Cabildo and Petorca, network theory suggests consensual decision making might be more difficult in this situation, and may have led some APRs not to respect internal agreements. In both localities, those actors receiving stronger legitimacy by their peers were either external organizations or MOP-APRs because they either disposed or had access to resources that the non-MOP-APRs lacked. Apart from these commonalities, we observed considerable differences in the legitimacy networks between the two Communal Unions.

**Fig. 3:**
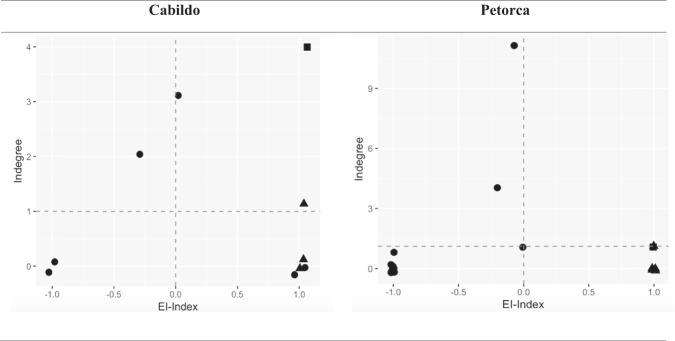
Empirical distribution of legitimacy in the networked lateral governance matrix. Note: Circles = MOP-APRs; triangles = non-MOP-APRs; squares = external organizations

Figure 3 shows the empirical distribution of legitimacy according to the legitimacy matrix, which displays the structure of legitimacy on the horizontal and the strength of legitimacy of an actor on the vertical axis (Glückler 2020). The structure of legitimacy measures the extent to which a member is found legitimate either from within their own or by other subgroups of the network. The upper half of the matrix displays actors with above-average strength in legitimacy as measured by indegree. Whereas the upper left quadrant represents strongly legitimate actors with local legitimacy (EI-Index below zero), the upper right quadrant shows strongly legitimate actors with global legitimacy (positive EI-Index). Globally legitimate actors source their legitimacy from distinct factions or interconnected sub-clusters and so bridge the potential gaps between the factions. Actors with an EI-Index close to zero have as many external as internal relations, so they receive legitimacy from their home faction (local legitimacy) as well as from other factions (global legitimacy).

In the case of Cabildo, the network centralization of legitimacy was 0.24, indicating relative dispersion of legitimacy relations (Fig. 2). Many organizations did not trust other members enough to entitle them to represent their point of view in decision making. The single only actor with global legitimacy was part of an external organization that did neither belong to an APR (Fig. 2; actor P, in blue), nor to the territory of Cabildo. This organization was also the most frequently named partner in collaboration by all other members. This high centrality of a non-member had severe implications for the Communal Union. First, there are only 16 leaders in Cabildo, counting the external actor. In the hypothetical question of delegation of legitimacy, six leaders stated that they did not trust anyone in the communal Union to make decisions for them. Second, the stability of Cabildo’s network governance was fragile, at least more fragile than in Petorca, because the whole structure of collaboration and legitimacy depended on a single actor who was not part of the local governance structure and therefore left a vacuum if this actor left the Communal Union. Thirdly, the observed network governance differed from the planned network governance structure (Glückler 2020) because the governance design would predict that legitimacy would be concentrated on members of the Communal Union rather than on non-members (Fig. 3). This also suggests that lateral water governance, at the empirical level, is complex, and is not governed by a planned and/or formal structure.

In the case of Petorca, while having the same network density, the overall centralization of the legitimacy network was twice as high as in Cabildo, suggesting that legitimacy was concentrated in a few actors who were all active members of the Communal Union except for one. Three of the largest members were also the most collaborative as well as the most legitimate actors (Fig. 2; actors J, E and U, in yellow). In addition, one non-MOP-APR and an external organization (Office of Water Affairs) enjoyed global yet only moderately strong legitimacy. Three MOP-APRs enjoyed strong yet moderately local global legitimacy (EI-index approx. zero). A MOP-APR that corresponds to a large cooperative supplying drinking water to a large number of families was the one with the greatest local and global legitimacy. In this sense, the structural patterns of the legitimacy matrix in Petorca reveal that nine people delegated decision-making power to the leader of this MOP-APR. Its leader was recognized in the interviews as very committed and active. Whereas the theory of lateral governance suggests that governance effectiveness rises with global legitimacy (Glückler 2020), we add a dynamic component to the legitimacy matrix by arguing that the most legitimate member who actually enjoys a balanced legitimacy (EI-Index = 0) had not yet acquired greater global legitimacy due to the embryonic stage of the organization. Because the legitimacy matrix produces only a snapshot of the current situation, we cannot directly infer the inherent dynamism of how legitimacy evolves and spreads in governance network over time. Moreover, because there is a difference in resources between MOP-APRs and non-MOP-APRs, the Petorca Communal Union has been more committed to collaboration to compensate for the lack of state support and meet the most urgent needs of the most fragile non-MOP-APRs. Accordingly, most of the leaders of the non-MOP-APRs delegated legitimacy to MOP-APR leaders. These dynamics explain the fact that the most active and legitimate members occupy a network position of balanced legitimacy.

In summary, a comparison of the quality of network governance activities and structure, in terms of collaboration and legitimacy, shows that these factors are consistent with greater effectiveness in governance outcomes even when they are in still immature states of network governance. The conjecture that a particular type of network structure produces the same outcome is often a fallacy (e.g., Rowley et al. 2000). Whereas a particular network structure may have certain outcomes in a particular context or time period, it may have different effects in others (Glückler and Panitz 2021). As a consequence of this structural contingency, we prefer to argue for coherence rather than causality of conditions. Conditions are considered coherent if one condition reinforces the other (Boyer 2005). Hence, we reject notions of structural determinism and argue that in the context of our case study, a more diversely composed core of the collaboration as well as of the legitimacy network, and a more strongly interconnected Union with external partners through bridging organizations were strongly coherent with the higher effectiveness of governance outcomes in the Petorca Union. Our focus on the APRs’ ability to raise public funds necessary to build physical and organizational infrastructure may be seen as only a partial measure of governance effectiveness and, hence, a limitation. A more comprehensive analysis would also include other achievements, such as capacity building, the quality and magnitude of water provision to all users, the quality of the physical infrastructure, etc. These data were, however, not available in this study.

## Conclusions

Climatic, economic, and political factors widely threaten water security in rural areas. Whereas hierarchical and centralized organizations have focused on technological efficiency and have tended to disregard regional variation and territorial contextuality, effective water governance requires more lateral coordination between local water users and associations as well as better connectivity across geographical and hierarchical scales. Processes of decentralization have enabled bottom-up networks to establish and to take collective action and adapt more flexibly to particular local conditions.

The comparative assessment of two newly formed collective governance entities in Petorca and Cabildo has shown that at the local level, relational characteristics of collaboration and legitimacy are essential to improve effective water governance. Even in a context of high vulnerability, some APRs have mobilized resources, new practices, and knowledge to meet the common objective of providing water to the community without formal support by the state. Despite similar political, economic, social and cultural, and geographical conditions, the two Communal Unions developed distinct relational qualities in terms of activities and network structure. We found that these differences in the structure of legitimacy and collaboration were coherent with the differences in governance performance as measured by the ability of APRs to raise subsidies.

Much remains to be understood about what relational characteristics may be conducive to achieving adequate governance in specific water scarcity contexts. A relational assessment of collaboration and legitimacy and their distribution within and across governance networks is recommended as a helpful diagnostic for both researchers and practitioners in resource governance. Understanding the structure of these relations raises the potential for consensus, adaptation, and realization of the ultimate goal of securing water supply for peripheral rural populations.

Finally, it is necessary to emphasize that this case study only represents the local level of interaction patterns. Yet these local water governance entities are only a part of a more complex polycentric system of water governance, where local actors still depend strongly on resources and capacities provided by public authorities and administrative bodies at local, regional and national levels. Hence, the scarcity of drinking water is a much broader and systemic environmental and societal challenge that requires legislative changes to ensure water availability through prioritization of uses and organizational and legislative autonomy of communities to manage drinking water effectively.

## Footnotes

1 A Communal Unions is a territorial organization formed by the leaders of local rural drinking water associations within the corresponding territory to improve the representativeness of the sector. Within the region Valparaíso, the provinces of Petorca, Cabildo, and La Ligua had established Communal Unions at the moment of conducting this study.
